# Toward Precision Radiotherapy: A Nonlinear Optimization Framework and an Accelerated Machine Learning Algorithm for the Deconvolution of Tumor-Infiltrating Immune Cells

**DOI:** 10.3390/cells11223604

**Published:** 2022-11-14

**Authors:** Lois Chinwendu Okereke, Abdulmalik Usman Bello, Emmanuel Akwari Onwukwe

**Affiliations:** 1 Department of Pure and Applied Mathematics, Mathematics Institute (Emerging Regional Centre of Excellence (ERCE) of the European Mathematical Society (EMS)), African University of Science and Technology, Abuja 900107, Nigeria; 2 Department of Mathematics, Federal University Dutsin-Ma, Dutsin-Ma 821101, Nigeria; 3 Department of Theoretical and Applied Physics, African University of Science and Technology, Abuja 900107, Nigeria; 4 Inspired Innovative Sustainable (IIS) Projects & Solutions Limited, Abuja 900107, Nigeria

**Keywords:** predictive biomarkers, bulk RNA-seq, nonlinear regression, inverse problem, digital cytometry, bioinformatics, immune contexture, nonlinear functional analysis, constrained optimization, error analysis

## Abstract

Tumor-infiltrating immune cells (TIICs) form a critical part of the ecosystem surrounding a cancerous tumor. Recent advances in radiobiology have shown that, in addition to damaging cancerous cells, radiotherapy drives the upregulation of immunosuppressive and immunostimulatory TIICs, which in turn impacts treatment response. Quantifying TIICs in tumor samples could form an important predictive biomarker guiding patient stratification and the design of radiotherapy regimens and combined immune-radiation treatments. As a result of several limitations associated with experimental methods for quantifying TIICs and the availability of extensive gene sequencing data, deconvolution-based computational methods have appeared as a suitable alternative for quantifying TIICs. Accordingly, we introduce and discuss a nonlinear regression approach (remarkably different from the traditional linear modeling approach of current deconvolution-based methods) and a machine learning algorithm for approximating the solution of the resulting constrained optimization problem. This way, the deconvolution problem is treated naturally, given that the gene expression levels of pure and heterogenous samples do not have a strictly linear relationship. When applied across transcriptomics datasets, our approach, which also allows the coupling of different loss functions, yields results that closely match ground-truth values from experimental methods and exhibits superior performance over popular deconvolution-based methods.

## 1. Introduction

In recent times, the continuous rise in the global cancer burden has emphasized the need for increased efforts in cancer treatment strategies. In 2020 alone, there were 19.3 million new cases and almost 10 million cancer deaths worldwide, with the number of new cases projected to climb to 28.4 million by 2040 [1]. This projection is not far-fetched, given the 36.9% increase in the number of new cases from 2012 to 2020 [2]. With up to half of all these cancer cases receiving radiotherapy during their treatment, radiotherapy is still a vital cancer treatment strategy [3,4,5]. 

In simple terms, radiotherapy involves using ionizing radiation, usually high-energy X-rays, to kill cancer cells or, at least, limit their proliferation by damaging their genetic code of life, known as deoxyribonucleic acid (DNA). In doing this, the goal is to achieve tumor control without introducing severe damage to surrounding normal tissues, enhancing treatment outcomes and minimizing adverse effects. Precision radiotherapy aims to reach this goal by stratifying and precisely treating “each individual cancer patient, using state-of-the-art new radiotherapy technology and biomarkers” [6].

Biomarkers are objectively evaluated and measured characteristics indicative of normal (or abnormal) biological processes, pathogenesis, or therapeutic response [7]. Their roles could be prognostic, diagnostic, treatment response monitoring, or predictive [8]. In their predictive role, biomarkers indicate the likelihood of a therapeutic benefit from a specific treatment for a given patient. Thus, in the case of precision radiotherapy, the complementary role of biomarkers for predictive purposes evolved from recent findings, showing that the effects of radiotherapy on the tumor microenvironment (TME) may be a significant determinant of the efficacy of a radiotherapy regimen [8,9,10]. For instance, in addition to damaging the malignant part of the TME, radiotherapy has been found to trigger immunomodulatory effects and alterations to critical components of the TME, such as tumor-infiltrating immune cells (TIICs) [9,11,12,13]. The latter is of particular interest because, as radiotherapy drives the upregulation of immunostimulatory TIICs such as cytotoxic CD8+ T cells, and immunosuppressive TIICs such as regulatory T cells (Treg), its impact is felt on differing cell subsets [13]. Consequently, quantifying TIICs in pretreatment and treatment of tumor samples is crucial in identifying predictive biomarkers guiding patient stratification and designing suitable radiotherapy regimens, including combined immune-radiation treatments [14].

Traditionally, experimental methods such as immunohistochemistry (IHC), cytometry, and recently, single-cell RNA sequencing (scRNA-seq) have been the gold standard for quantifying TIICs in samples. Although these methods precisely quantify TIICs in samples, there are limitations in terms of the technicality and range of applicability associated with each method. On the one hand, scRNA-seq is not just expensive and laborious for routine use but also highly prone to bias due to variations in the dissociation efficiencies of single cells [15]. On the other hand, IHC and cytometry rely on a small number of phenotypic markers, exhibit low to medium throughput, have little or no public datasets available, and are difficult to apply in large tumor series [16]. This situation necessitates the search for suitable alternatives to quantify TIICs in tumor samples.

Recently, the sharp reduction in the cost of next-generation sequencing (NGS) technologies has encouraged its routine application in clinical settings, resulting in the availability of large amounts of transcriptomics datasets from patients’ tumor samples, such as The Cancer Genome Atlas (TCGA) [17]. Although these datasets represent the bulk tumor sample, they provide a suitable alternative for quantifying the sample TIICs using computational techniques [18]. Computational techniques serving this purpose are broadly categorized into two. The first broad category is marker gene-based methods [14,19]. Methods under this category utilize a list of genes characteristic of a cell type (called marker genes), to quantify every cell type independently from the expression levels of the marker genes in the heterogenous tumor sample. As a result, marker gene-based methods can only generate “a semi-quantitative score describing the enrichment of a cell type in a sample” [14], thus effectively making the comparison between cell types impractical.

The second broad category, deconvolution-based methods, considers the gene expression profile of the heterogeneous tumor sample as a *convolution* of the gene expression levels of the different cell components [20]; as a result, they can quantitatively estimate the fractions of cell types of interest (in this case TIICs). This consideration allows the problem to be formulated mathematically as a function of the gene expression profiles of the cell-type admixture. Thus, given the bulk gene expression of a tumor sample and a known cell-type specific expression profile, solving an inverse problem can estimate the cell-type fractions in the heterogeneous tumor sample. 

To date, most deconvolution-based methods, including those specific to quantifying TIICs, such as CIBERSORT [21], CIBERSORTx [22], EPIC [23], ECIS [24], quanTIseq [25], and TIMER [26], assume that function to be linear. However, they yield different results for different cell types and use cases despite utilizing the gradient algorithm or its variants to approximate solutions to the inverse problem. Interestingly, this is because each method is conceptually different according to the choice of loss function (or objective functions) and the setting of the optimization problem (constrained or unconstrained). It is against this backdrop that packages such as Immunedeconv [27], TIMER2.0 [28], and TumorDecon [29] have sought to provide a unified platform that allows each of the different methods to be applied on the same dataset to compare or complement results. Accordingly, the strengths of each method can be harnessed to gain more robust and comprehensive estimates. Nevertheless, this approach is still susceptible to the potential issues associated with traditional linear modeling [30], given that “the relationship between the expression levels of pure and heterogeneous samples is not strictly linear” [14]. Moreover, dealing with large transcriptomics datasets calls for computationally efficient methods with fast rates of convergence and runtime [30].

Therefore, the main aim of this paper is to introduce and discuss a mathematical formulation that permits the TIIC deconvolution inverse problem to be handled in its natural state alongside an accelerated machine learning algorithm for approximating its solution. Through rigorous mathematical analysis, we show that the algorithm converges to an optimal solution of the inverse problem for various loss functions. More specifically, a globally optimal solution is guaranteed for convex loss functions. Furthermore, we use numerical experiments to show that the algorithm exhibits faster convergence rates and runtime than other traditionally used algorithms. When applied across transcriptomics datasets, our results closely match values from experimental methods and show superior performance over popular TIIC deconvolution-based methods. We end with a note on the detailed science behind these observations and an explanation of how this framework can be applied across similar inverse problems in biology, medical physics, and oncology.

## 2. Materials and Methods

### 2.1. Formulation and Discussion of the Deconvolution Problem

Let N denote the number of different cell types forming a mixture sample, and M be the number of genes whose expressions are measured in the sample. Let $B=\left(b_{1},\;b_{2}\ldots ,b_{M}\right)\in {\mathbb{R}}^{M}$ be the measurements of gene expression in the sample. Let $S\in {\mathbb{R}}^{M\times N}$ be the corresponding reference expressions matrix of the M genes from the N constituent cell types, and $P=\left(p_{1},\;p_{2}\ldots ,p_{N}\right)\in {\mathbb{R}}^{N}$ be the unknown proportions of mix of the different cell types. An operator r can model the relationship of B, S, and P as

(1) $$B=r\left(S,P\right).$$

The deconvolution problem is concerned with the inverse problem of estimating P, given B and S. This inverse problem can be formulated mathematically as the following equivalent constrained optimization problem:

(2) $$\underset{P\in C}{min}L\left(B,r\left(S,P\right)\right),$$

where L is a loss function measuring model fitness, and C is the set of constraints on P arising naturally from its definition as proportions, i.e.,

$$C=\left\{P\in {\mathbb{R}}^{N}:\;p_{i}\geq 0\;\forall \;i=1,\ldots ,N,\;\overset{N}{\underset{i=1}{\sum }}p_{i}=1\;\right\}.\;$$

Many of the existing deconvolution methods (see, for example, [21,22,23,31,32,33,34,35] and references therein) consider B, S, and P to be linearly related in the form

(3) B=SP+e, 

where e is a random error. Several issues with the linear framework have been identified [30]. More recently, the authors of [36] showed problems associated with different scales of gene expression within the linear framework and then proposed the following hybrid model:

(4) $$\log\left(b_{i}\right)=d+\log\left(\overset{N}{\underset{j=1}{\sum }}S_{ij}p_{j}\right)+e_{i},\;$$

where d accounts for systemic technical variation. Equivalently,

(5) $$B\approx exp\left(d\right)SP.\;$$

We note that $\exp\left(d\right)$ in Equation (5) is a constant factor adjustment across all genes. However, more than this constant factor adjustment may be required, because such systemic technical variations affect genes differently [37]. Thus, one may consider a more general model of the form

(6) B≈DSP,

where D is a diagonal matrix with diagonal entries as gene-specific factor adjustment generated from some known distribution. Accordingly, Equations (3) and (5) become special cases of Equation (6). Nevertheless, such a linear transformation may not efficiently describe nonlinear patterns. 

Consequently, we formulate a generic nonlinear framework for the deconvolution problem by considering the operator r in Equation (1) as a nonlinear transformation involving S and *P*. This is because generalized nonlinear regression methods have been shown to yield better prediction accuracy for complex nonlinear patterns, where traditional linear regression models may fail [38]. Specifically, our nonlinear operator r is given as

(7) $$r_{i}={\left(\delta +\overset{N}{\underset{j=1}{\sum }}S_{ij}p_{j}\right)}^{\theta },\;\delta ,\theta >0.$$

The nonlinearity of Equation (7) depends on the value of θ. Note that Equation (7) reduces to the linear framework for θ=1. Therefore, for an arbitrary sequencing dataset, it is worthwhile to determine what values of θ (different from one) closely describe the not strictly linear relationship of the gene expression profiles of pure and heterogeneous samples. 

Rigorous data assimilation techniques are useful in making such determinations from arbitrary datasets. However, we refrain from such nontrivial rigorous analytical examinations as they are beyond the aims of this work. As a result, we choose our θ values using an empirical approach for the purpose of demonstrating the proof of principle which is the subject of this work. The empirical approach is based on our hypothesis that, for some $\theta \in \left(0,1\right)\cup \left(1,2\right)$, we may be able to get suitable values satisfying the description of the relationship between the gene expression profiles of pure and heterogeneous samples. More specifically, we hypothesize that such a value might be slightly less than one or slightly greater than one on the order of a few decimal places. This hypothesis is guided by our preferred interpretation of the expression “not strictly linear”.

### 2.2. Review of Some Commonly Used Loss Functions

Several research surveys of cell-type deconvolution methods (see, for example, [14,19,39]) have identified the quadratic/squared error loss and the ε-insensitive loss, as the most commonly used loss functions for reference-based cell-type deconvolution within the linear framework. On the one hand, the squared error loss (SEL) is formulated on the basis of squared deviations as follows:

(8) $$L\left(B,r\left(S,P\right)\right)=\overset{M}{\underset{i=1}{\sum }}{\left(b_{i}-S_{i}P\right)}^{2}=B-SP_{2}^{2},$$

where $S_{i}$ denotes the *i-*th row of S. In fact, it is the most common choice of loss function due to its simplicity. Notably, the SEL is highly susceptible to outliers. This feature can be especially beneficial when the outliers originate naturally from variations within the process and, as such, contain useful systemic information. Conversely, it can be a drawback when the outliers arise from noise (errors).

On the other hand, the ε-insensitive loss (also referred to as the support vector method) [40] and other robust techniques, such as the Huber and Laplacian losses, are employed to reduce the drawback of the SEL. A unified version of these robust techniques was introduced in [41] as a soft insensitive loss function (SILF), expressed mathematically as

(9) $$L\left(B,r\left(S,P\right)\right)=\overset{M}{\underset{i=1}{\sum }}l_{i}\left(b_{i}-S_{i}P\right),$$

where

$$l_{i}\left(a\right)=\left\{\begin{array}{c} -a-\varepsilon ,\;if\;a\in \left(-\infty ,-\left(1+\rho \right)\varepsilon \right)\\ \begin{array}{c} \frac{{\left(a+\left(1-\rho \right)\varepsilon \right)}^{2}}{4\rho \varepsilon },\;if\;a\in \left[-\left(1+\rho \right)\varepsilon ,-\left(1-\rho \right)\varepsilon \right]\;\\ 0,\;if\;a\in \left(-\left(1-\rho \right)\varepsilon ,\left(1-\rho \right)\varepsilon \right) \end{array}\\ \begin{array}{c} \frac{{\left(a-\left(1-\rho \right)\varepsilon \right)}^{2}}{4\rho \varepsilon },\;if\;a\in \left[\left(1-\rho \right)\varepsilon ,\left(1+\rho \right)\varepsilon \right]\\ a-\varepsilon ,\;if\;a\in \left(\left(1+\rho \right)\varepsilon ,+\infty \right) \end{array} \end{array},\right.\;$$

with 0<ρ≤1 and ε>0. In [41], the authors remarked that this function (Equation (9)) is smooth and inherits most of the desirable characteristics of several robust techniques, including insensitivity to outliers. They further demonstrated in great detail the computational efficiency and competitiveness of SILF compared to other well-respected techniques. Remarkably, these loss functions have been a dominating paradigm in the deconvolution problem, mainly due to their convexity.

Nevertheless, it has been shown in recent times that nonconvex loss functions improve the generic applicability and robustness of learning, especially in situations where the data and noise distributions are unknown [42]. One such loss function is given in Equation (10) as a Cauchy kernel risk-sensitive loss (CKRSL), derived using a Gaussian kernel-adapted operator and following methods similar to those in [43,44].

(10) $$L\left(B,r\left(S,P\right)\right)=\frac{1}{M}\overset{M}{\underset{i=1}{\sum }}\beta \log\left[1+2\left(\frac{1-\exp\left(-{\left(\frac{\sum_{j=1}^{N}S_{ij}p_{j}-b_{i}}{2\sigma }\right)}^{2}\right)}{\beta }\right)\right],\;\sigma >0.$$

We remark that the abstract formulation presented in Section 2.1 has the advantage of accommodating several loss functions including those reviewed in this subsection. 

### 2.3. Specification of the Loss Function for this Study

For the present study, we use a weighted squared error loss (WSEL) on the relationship operator r defined by Equation (7), expressed as

(11) $$L\left(B,r\left(S,P\right)\right)=\frac{1}{M}\overset{M}{\underset{i=1}{\sum }}{\left({\left(\delta +\overset{N}{\underset{j=1}{\sum }}S_{ij}p_{j}\right)}^{\theta }-b_{i}\right)}^{2}.$$

### 2.4. Accelerated Machine Learning Algorithm (AMLA)

An optimal solution to the constrained optimization problem of Equation (2) can be approximated by the following accelerated machine learning algorithm (AMLA):

(12) $$\left\{\begin{array}{c} v_{0},v_{1}\;\in \;C\\ w_{j}=v_{j}+\alpha_{j}\left(v_{j-1}-v_{j}\right)\\ v_{j+1}={℘}_{C}\left(w_{j}-\lambda Av_{j}\right) \end{array},\right.$$

for some predefined values of the adaptive momentum parameter $\alpha_{j}$ and step size (or learning rate) λ. A denotes the gradient of the desired loss function L, and ${℘}_{C}$ is the projection operator on C. The projection operator ${℘}_{C}$ locates a point in C having the least distance to a given point, while $v_{0},v_{1}\;$are initialization points. 

AMLA converges to a solution of the deconvolution problem (Equation (2)) for various loss functions, including those reviewed in Section 2.2. A detailed mathematical analysis establishing this convergence is presented in Appendix A, starting with the preliminary mathematical tools, as well as the lemmas, theorems, and their proofs. 

For our choice of loss function (Equation (11)), the gradient A is the vector defined by

(13) $$A_{j}=\frac{2\theta }{M}\overset{M}{\underset{i=1}{\sum }}S_{ij}\left({\left(\delta +\overset{N}{\underset{k=1}{\sum }}S_{ik}p_{k}\right)}^{\theta }-b_{i}\right){\left(\delta +\overset{N}{\underset{k=1}{\sum }}S_{ik}p_{k}\right)}^{\theta -1}.$$

Furthermore, the control parameters $\alpha_{j}$ and λ are determined by a Lipschitz constant (K) of A (See Theorem 12 in Appendix A). For A defined by Equation (13), a suitable K can be given by

(14) $$K=\left\{\begin{array}{c} N\left[\frac{2\theta^{2}s_{\max}^{2}}{\delta^{2(1-\theta )}}+\frac{2\theta |\theta -1|s_{\max}^{2}\left[{\left(Ns_{\max}+\delta \right)}^{\theta }+b_{\max}\right]}{\delta^{2-\theta }}\right],\;\mathrm{for}\;\theta \leq 1\\ N\left[2\theta^{2}s_{\max}^{2}{\left(Ns_{\max}+\delta \right)}^{2(\theta -1)}+\frac{2\theta |\theta -1|s_{\max}^{2}\left[{\left(Ns_{\max}+\delta \right)}^{\theta }+b_{\max}\right]}{\delta^{2-\theta }}\right]\;\mathrm{for}\;1<\theta \leq 2\\ N\left[2\theta^{2}s_{\max}^{2}{\left(Ns_{\max}+\delta \right)}^{2(\theta -1)}+2\theta \left|\theta -1\right|s_{\max}^{2}\left[{\left(Ns_{\max}+\delta \right)}^{\theta }+b_{\max}\right]{\left(Ns_{\max}+\delta \right)}^{\theta -2}\right],\;\mathrm{for}\;\theta >2 \end{array},\right.$$

where $s_{max}=\underset{\begin{array}{c} 1\leq i\leq M\\ 1\leq j\leq N \end{array}}{\max}\left|S_{ij}\right|,\;b_{max}=\underset{1\leq i\leq M}{\max}b_{i}.$

Lastly, the projection operator on C denoted by ${℘}_{C}$ is computed using the alternating projections method [45]. 

### 2.5. Validation Datasets

Our validation datasets, which come from published findings in [22,23], consisted of experimentally measured immune cell-type proportions from tumor samples, bulk RNA sequencing data of tumor samples, and a gene expression reference profile. The gene expression reference profile was a signature matrix of eight Melanoma subsets derived from scRNA-seq (SMART-Seq2) (see Supplementary Table S2e in [22]). These melanoma subsets are listed across 3121 genes. They include five TIICs (B cells, CD8 T cells, CD4 T cells, NK cells, and macrophages) and other cell subsets, including endothelial cells, malignant cells, and cancer-associated fibroblasts (CAFs).

Experimentally measured immune cell-type proportions from tumor samples and bulk RNA sequencing data of tumor samples were obtained from [23]. In [23], the authors divided the single-cell suspensions collected from the lymph nodes of four patients with metastatic melanoma into two portions. For one portion, flow cytometry was used to measure the percentages of live cells, including four TIICs (B cells, CD4 T cells, CD8 T cells, and NK cells), malignant cells, and other cells made up of primarily stromal and endothelial cells (see Supplementary Table S3A in [23]). The fractions of each of these cell types for each patient are presented in Table 1 below. We refer to these cell-type fractions as the ground-truth values from the experiment (GTVEs).

The other portion of the single-cell suspensions was used for bulk RNA sequencing (RNA-seq). We downloaded this RNA-seq data for the four Melanoma patients from the “example data from EPIC” link on the EPIC web application (http://epic.gfellerlab.org) accessed on 28 September 2022. It can also be accessed from the Gene Expression Omnibus (GEO) repository [46] through the accession number GSE93722. This RNA-seq data consists of 49,902 genes quantified in transcripts per million (TPM) for each of the four melanoma samples.

### 2.6. Deconvolution Workflow

**Figure 1:**
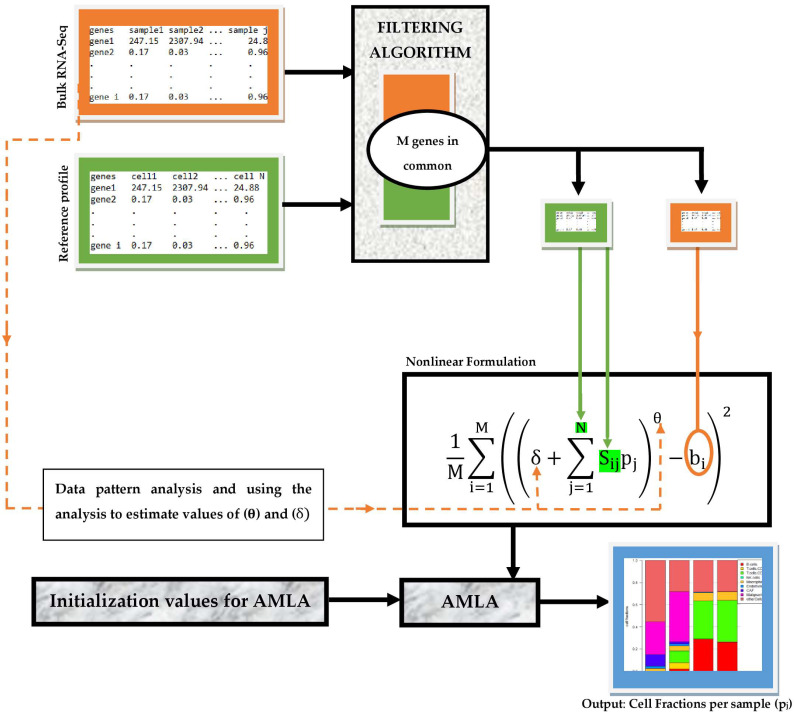
Workflow for the deconvolution of TIICs using a nonlinear optimization framework.

Our deconvolution workflow consists of partly sequential steps necessary to achieve efficient and accurate estimation of TIICs from bulk RNA-seq data. As illustrated in Figure 1, the input data comprise a tab-delimited text file of bulk RNA-seq samples and gene reference profiles. These inputs are first processed using a simple data filter algorithm, which identifies the genes common to both inputs and passes values of these genes in each input data across the filter. These values are then fed into the respective variables of our nonlinear framework. After that, the cell fractions per sample are computed using AMLA. We remark that suitable values of the parameters *θ* and *δ* can be estimated using a rigorous non-trivial pattern analysis of the bulk RNA-Seq data, as indicated in Figure 1, although no attempt was made in this direction in the present work. 

### 2.7. Software Used

We implement the deconvolution workflow described in Figure 1 as a Python package. The package was developed using the IDE (Integrated Development Environment) PyCharm Community Edition 2021.2.1 version 212.5080.64 created by JetBrains s.r.o, Prague, Czech Republic, running Python 3.9.7 (64 bit) version 3.9.7150.0. The package contains three custom-made modules: the filtering algorithm, our nonlinear framework, and AMLA. The Pandas library was used to manipulate the import of sequencing data and reference profiles and export of estimated fractions for visualization. 

To compare our method with two popular cell-type deconvolution methods, we also generated results from CIBERSORTx [22] and EPIC [23] using the web application versions of their software available at https://cibersortx.stanford.edu/ accessed on 30 September 2022 and http://epic.gfellerlab.org accessed on 28 September 2022, respectively. We performed all these software activities on an Intel^®^ Core™ i5-6300U CPU @at 2.40GHz with 8 GB RAM on a 64 bit Windows 10 Pro operating system.

## 3. Results

### 3.1. Estimating Cell-Type Fractions in Four Melanoma Samples Using Our Nonlinear Framework, EPIC, and CIBERSORTx

We considered the bulk RNA-Seq dataset of four melanoma patients and the gene reference profile containing eight cell subsets, as described in Section 2.5. By inputting tab-delimited text files of these two datasets into the filtering algorithm, we obtained 2928 genes common to both datasets. The values for these specific genes in the respective datasets were passed across the filtering algorithm into the nonlinear framework. We estimated eight cell-type fractions present in each of the four melanoma samples using four different versions of our nonlinear framework. These versions, named according to the value of the hyperparameter θ in Equation (11) and the procedure for applying AMLA, include those described below.

1. Equivalent linear model (ELM), expressed for θ=1, such that Equation (11) becomes

(15) $$L\left(B,r\left(S,P\right)\right)=\frac{1}{M}\overset{M}{\underset{i=1}{\sum }}{\left(\left(\delta +\overset{N}{\underset{j=1}{\sum }}S_{ij}p_{j}\right)-b_{i}\right)}^{2}.\;$$

AMLA is then applied to approximate the solution.

2. Linearized nonlinear model (LNM), expressed for θ=0.92, such that Equation (11) becomes

(16) $$L\left(B,r\left(S,P\right)\right)=\frac{1}{M}\overset{M}{\underset{i=1}{\sum }}{\left({\left(\delta +\overset{N}{\underset{j=1}{\sum }}S_{ij}p_{j}\right)}^{0.92}-b_{i}\right)}^{2}\;.$$

However, we do not apply AMLA directly to Equation (16). Rather, we linearize it to obtain the form

(17) $$L\left(B,r\left(S,P\right)\right)=\frac{1}{M}\overset{M}{\underset{i=1}{\sum }}{\left(\left(\delta +\overset{N}{\underset{j=1}{\sum }}S_{ij}p_{j}\right)-{\left(b_{i}\right)}^{\frac{1}{0.92}}\right)}^{2},\;$$

and thereafter apply AMLA to approximate the solution.

3. Nonlinear model one (NM1), expressed for θ=0.92, such that we obtain Equation (16) above, and then apply AMLA to approximate the solution.
4. Nonlinear model two (NM2), expressed for θ=1.08, such that Equation (11) becomes

(18) $$L\left(B,r\left(S,P\right)\right)=\frac{1}{M}\overset{M}{\underset{i=1}{\sum }}{\left({\left(\delta +\overset{N}{\underset{j=1}{\sum }}S_{ij}p_{j}\right)}^{1.08}-b_{i}\right)}^{2}.$$

AMLA is then applied to approximate the solution.

For all four versions enumerated above, we set the variable δ, such that δ=1. Moreover, we initialized AMLA with distinct set values of $v_{0}\;$and $v_{1}$, creating a cartesian product for the patient series. Table 2 summarizes the hyperparameter values δ and θ chosen for the named versions of our nonlinear framework.

We have already emphasized that the nonlinearity of our model (Equation (11)) strictly depends on the value of θ, which must be different from one. Furthermore, we remarked that the selection and tuning procedure for this parameter can be achieved analytically on the basis of the input gene sequencing datasets using rigorous data assimilation techniques. However, our primary goal in this work was to demonstrate that a nonlinear regression approach for the TIIC deconvolution problem could yield significantly more accurate estimates of the fractions of cell types, including TIICs from the bulk gene expression data of tumor samples. Thus, we favored an empirical approach for selecting the hyperparameter θ, in line with this goal. 

Our empirical approach relies on the observation that the relationship between the gene expression profiles of pure and heterogeneous samples is not strictly linear. Guided by this, we interpret the expression “not strictly linear” as slightly different from one, and this difference can be either side of one, i.e., greater than or less than one. Because θ is strictly positive, we looked at $\theta \in \left(0,1\right)\cup \left(1,+\infty \right)$. Guided by our hypothesis presented in Section 2.1, we randomly selected 0.92 from the interval $\left(0,1\right)$. We also selected 1.08 from the interval $\left(1,+\infty \right)$ by considering the symmetric distance of the previous selection from one. The hyperparameter δ is a smoothing parameter. The literature is filled with several rigorous techniques for smoothing parameter estimation. Again, for the same reasons as in θ, we chose to assume a default value of one. 

We present two results when estimating the cell-type fractions in the four melanoma samples using EPIC. The first result which we denote as “EPIC1” was obtained from the default setting of the EPIC web application (http://epic.gfellerlab.org) accessed on 30 September 2022. The default setting comprises tab-delimited inputs of bulk RNA-seq dataset as described in Section 2.5. Furthermore, it includes a reference profile of seven cell subsets (B cells, CAFs, CD4 T cells, CD8 T cells, endothelial, macrophages, and NK cells), built from tumor-infiltrating cells from TPM normalized scRNA-seq (see Supplementary Table S2A in [23]). This reference profile contains 23,684 genes. The second result, which we denote as “EPIC2”, was obtained using the datasets as described in Section 2.5.

Furthermore, we estimated the cell-type fractions in the four melanoma samples using the CIBERSORTx web application (https://cibersortx.stanford.edu/) accessed on 30 September 2022. The gene expression reference profile used was as described in Section 2.5. Here, we present two results for our cell-type fractions estimation using CIBERSORTx. The first result, which we denote as “CIBERSORTx1”, was obtained by checking the batch correction box, selecting B-mode, and then running CIBERSORTx, after the tab-delimited bulk RNA-seq and reference profile files were uploaded. The second result, which we denote as “CIBERSORTx2”, was obtained following the same procedure as in the first result, with the only exception being to uncheck the batch correction box. In both estimations using CIBERSORTx, quantile normalization was disabled, and the permutation for significance analysis was set at 100. We present these results in Table 3.

It is easy to notice that many values for the deconvolution methods NM1 and NM2 were identical across Table 3. Although NM1 and NM2 had different θ values of 0.92 and 1.08, respectively, both θ values had a symmetric distance of 0.08 about 1.00. This observation directly suggests that the scalar θ (for θ≠1) in our nonlinear framework exhibits some symmetry about one, such that we can expect identical results for two choices of θ with the same symmetrical distance about 1.00.

### 3.2. Estimated vs. Experimentally Measured Cell-Type Fractions in the Four Melanoma Samples

**Figure 2:**
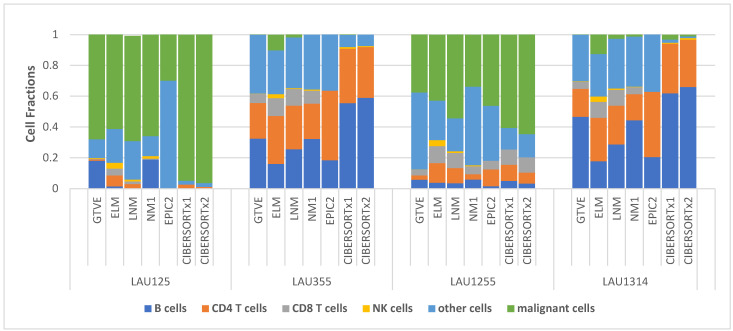
Stacked cell-type fractions per melanoma sample comparing results from different deconvolution methods with ground-truth experimental values.

We directly compare the estimated cell-type fractions in the four Melanoma samples presented in Table 3 with the ground-truth values from experiment (GTVE) presented in Table 1. To be able to do this, we ignored the EPIC1 results (due to the absence of malignant fractions) and NM2 results (as they are almost identical to NM1 results). Then, we aggregated the fractions—macrophages, endothelial cells, and CAFs—together as other cells since they satisfied the definition of other cells, as in Table 1. For EPIC2, we also added the values of “other cells” specified in the footnote below Table 3. The comparison is readily visualized in Figure 2. 

It is clear from Figure 2 that estimates from NM1 most closely matched the GTVE across all four samples, as indicated by the close resemblance of NM1 and GTVE stacks in all four samples. Even so, stacks from other versions of our nonlinear framework (ELM and LNM) appeared to more closely resemble the GTVE stacks in all four samples than the stacks from EPIC and CIBERSORTx. 

However, to quantitatively describe the extent of these resemblances, we considered a general-purpose error metric known as the root-mean-squared error (RMSE). RMSE is excellent for comparing the prediction error of different models for a specific variable. As a result, it is an incredibly good measure of model accuracy. The most accurate model would have an RMSE of zero, which is far from possible. Therefore, the model accuracy is determined by how close the RMSE is to zero, although this determination is made relative to the values of the observations or predictions. 

**Figure 3:**
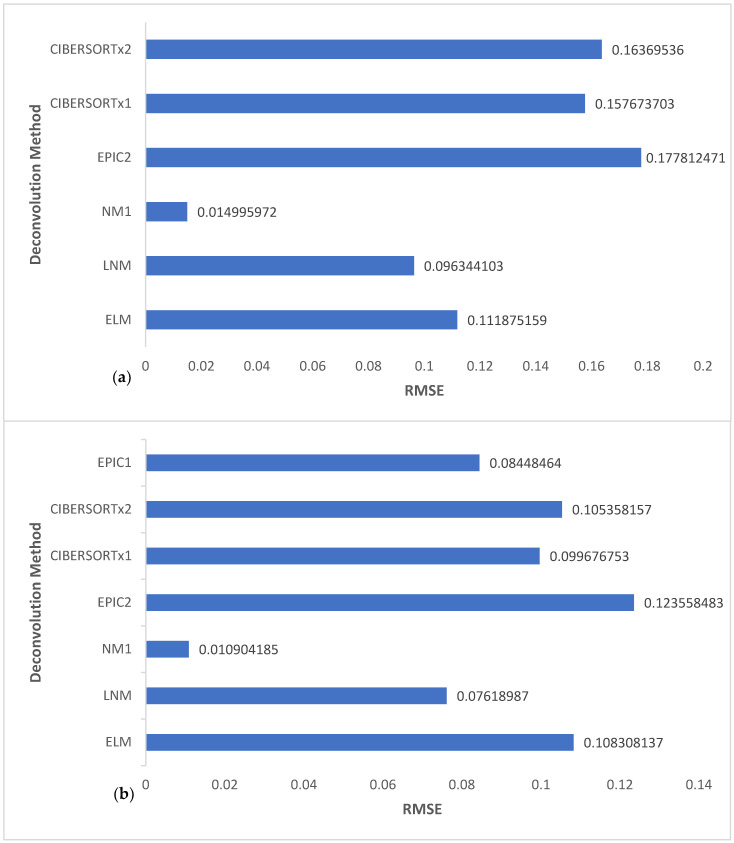
Comparison of the RMSE values of versions of the nonlinear framework, EPIC, and CIBERSORTx for (**a**) 24 observations including six cell subsets of the four melanoma samples, and (**b**) 16 observations including only the four TIICs of the four melanoma samples.

Here, we calculated the RMSE for NM1, LNM, ELM, CIBERSORTx1, CIBERSORTx2, and EPIC2 for all cell types of the four melanoma samples and then for TIICs only. In the latter, we also included the RMSE calculation for EPIC2. We present these results in Figure 3. We calculated the RMSE values using the formula

(19) $$\mathrm{RMSE}=\sqrt{\frac{\sum_{i=1}^{N}{\left(x_{i}-{\hat{x}}_{i}\right)}^{2}}{N}},$$

where N is the total number of analyzed cell-type subsets, $x_{i}$ is the GTVE of the analyzed cell-type subset, and ${\hat{x}}_{i}$ is the estimated value of the analyzed cell-type subset from a deconvolution method. Values from Table 1 and Table 3 were utilized in these calculations. 

NM1 can be seen to have the lowest RMSE in both charts of Figure 3. Remarkably, its RMSE value was significantly lower than that of all the other deconvolution methods compared and was extremely close to zero, being in the range of 0–0.02. This indicates that NM1 outperformed the other deconvolution methods and gave more accurate estimates that closely matched GTVE. The accuracy of NM1 can be directly attributed to the nonlinear framework used and the choice of the value of θ. Thus, we validated our hypothesis that a selection of θ slightly greater than or less than one on the order of a few decimal places accurately captures the not strictly linear sense of the relationship between the bulk gene expression and the reference profile. By using the same reference profile in the direct comparison of the deconvolution methods, we successful limited any chance that the results are a direct consequence of a factor other than the setting or framework of the deconvolution problem. 

## 4. Discussion

The linear framework has been the dominating paradigm in the computational deconvolution of TIICs and other tumor cell subsets from bulk gene expression data of tumor samples. Efforts toward improving the accuracy of cell fraction estimates from the computational deconvolution of bulk gene expression data have been centered on modifications still built around the basic linear framework. For instance, in both charts of Figure 3, CIBERSORTx1 had a slightly lower RMSE value when compared to CIBERSORTx2, indicating improved accuracy. Similar to the conclusion in [47], we attribute this improved accuracy to the batch correction effect in CIBERSORTx1, which aims to reduce data variability resulting from technical differences between samples. In another instance, in Figure 3b, EPIC1 had a lower RMSE value when compared to EPIC2, CIBERSORTx1, CIBERSORTx2, and ELM, possibly because of the use of a specific TIIC reference profile. On the other hand, in Figure 3a, ELM, a linear version of our nonlinear framework, had a lower RMSE value (more accuracy) when compared to EPIC2, CIBERSORTx1, and CIBERSORTx2. A likely reason is that the computational algorithm AMLA used in ELM projects directly onto the natural constraint set, which is much different from the computation in CIBERSORTx and EPIC, applied on some broadly defined constraints set, followed by a renormalization of the obtained values. 

It is clear from Figure 3 that these modifications would only yield minor improvements in accuracy since they are all based on the linear framework. Notably, the RMSE values from these linear framework-based models revolved around a narrow range of 0.11–0.18 and 0.08–0.11 in Figure 3a,3b, respectively. We may not expect any result different from these ranges if we were to analyze these datasets using other deconvolution methods, for example [24,25,26,31,32,33], because they are all based on the linear model with variations being in the choice of loss function or other technical modifications. 

As shown in Figure 3, the RMSE of NM1 is significantly low, and there is a remarkable difference between its RMSE range and that of the linear models. This observation demonstrates the enormous positive gains in terms of accuracy associated with modeling the deconvolution problem within a nonlinear framework, as it truly represents the natural state of the problem. We also emphasize through LNM results that any attempts to linearize the nonlinear framework before applying the solution algorithm (in this case, AMLA) would vastly diminish these positive gains. However, the outcome may still be slightly better than those from the traditional linear modeling. As evident from Figure 3, LNM RMSE values range from 0.07 to 0.10, which is significantly distant from the NM1 RMSE range (0–0.02) but much closer to the linear models’ RMSE ranges. For this reason, AMLA was especially designed to approximate solutions directly from the nonlinear framework without the need to linearize first. 

Furthermore, AMLA’s design allows it to exhibit faster rates of convergence and runtime in comparison to other traditionally used algorithms in machine learning, which is highly advantageous in the event of enormous amounts of bulk gene expression data of many tumor series. Verifying these with simple numerical experiments on ℝ (set of real numbers) using known loss functions employed in regression analysis is straightforward. We consider the log-hyperbolic loss, squared error loss, Cauchy loss, and ε-insensitive loss given, for example, by Equations (20)–(23), respectively.

(20) $$q\left(x\right)=\log\left(\cosh\left(6x-2\right)\right).$$

(21) $$q\left(x\right)={\left(5x-4\right)}^{2}.$$

(22) $$q\left(x\right)=5\log\left[1+\frac{{\left(3x-2\right)}^{2}}{5}\right].$$

(23) $$q\left(x\right)=\left\{\begin{array}{c} -x-\varepsilon ,\;if\;x\in \left(-\infty ,-\left(1+\rho \right)\varepsilon \right)\\ \begin{array}{c} \frac{{\left(x+\left(1-\rho \right)\varepsilon \right)}^{2}}{4\rho \varepsilon },\;if\;x\in \left[-\left(1+\rho \right)\varepsilon ,-\left(1-\rho \right)\varepsilon \right]\;\\ 0,\;if\;x\in \left(-\left(1-\rho \right)\varepsilon ,\left(1-\rho \right)\varepsilon \right) \end{array}\\ \begin{array}{c} \frac{{\left(x-\left(1-\rho \right)\varepsilon \right)}^{2}}{4\rho \varepsilon },\;if\;x\in \left[\left(1-\rho \right)\varepsilon ,\left(1+\rho \right)\varepsilon \right]\\ x-\varepsilon ,\;if\;x\in \left(\left(1+\rho \right)\varepsilon ,+\infty \right) \end{array} \end{array}.\right.$$

**Figure 4:**
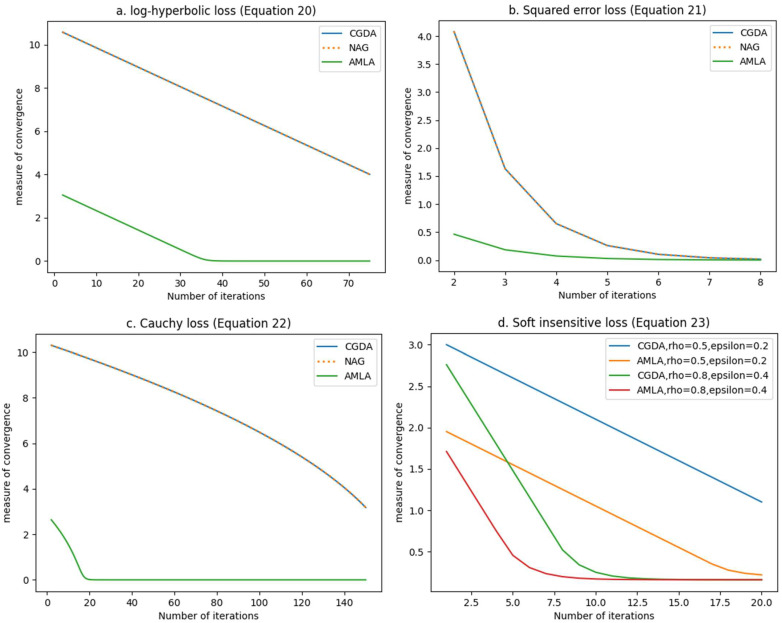
Comparison of the algorithmic efficiency of AMLA, NAG, and CGDA in approximating solutions for (**a**) log-hyperbolic loss (Equation (20)), (**b**) squared error loss (Equation (21)), (**c**) Cauchy loss (Equation (22)), and (**d**) ε-insensitive loss (Equation (23)).

For each of the Equations (20)–(23), we approximated their solutions using AMLA, the well-known classical gradient descent algorithm (CGDA), and Nesterov’s accelerated gradient (NAG) widely used in machine learning. Since the optimal solutions of the equations are known, we measured the convergence to the solutions from AMLA, CGDA, and NAG using variations of ${\left(x_{n}-\bar{x}\right)}_{n\in \mathbb{N}}$, where $x_{n}$ is the labeling obtained at the n-th iteration, and $\bar{x}$ is the optimal solution. We plot this measure of convergence against the number of iterations in Figure 4. 

From Figure 4, we can observe that AMLA converged in a significantly fewer number of iterations than CGDA and NAG, for all four loss functions considered. The implication of this observation is that AMLA has a higher order of convergence and, consequently, a faster rate of convergence since the “order of convergence defines the rate of convergence” [48]. Furthermore, Figure 4 affirms the robustness of AMLA. A robust algorithm is one that is theoretically guaranteed to converge, “starting from any initial design estimate” [49], “such that their correctness is not destroyed by round-off errors” [50]. We show in Appendix A, using rigorous mathematical analysis, that AMLA is guaranteed to converge to a solution of the constrained optimization problem (Equation (2)) for a variety of loss functions satisfying the stated conditions. The four loss functions presented in Figure 4 satisfy the stated conditions, thus leading to their convergence in Figure 4, even when CGDA and NAG did not converge (see Figure 4a,c). Moreover, as shown in Figure 4a–d, once AMLA converged, it maintained the flat zero line, which highlights its gross insensitivity to round-off errors, thus affirming its high robustness.

A very noteworthy affirmation of the high robustness of AMLA can be seen in Figure 4d. The ε-insensitive loss introduces extra parameters of ρ and ε whose selection can critically affect the convergence and robustness of any machine learning algorithm. As shown in Figure 4d, when we randomly selected ρ=0.5 and ε=0.2, AMLA converged in fewer than 20 iterations while CGDA was yet to converge beyond 20 iterations. Furthermore, when we randomly selected ρ=0.8 and ε=0.4, AMLA converged in about seven iterations, while CGDA converged in about nine iterations. These observations from Figure 4d clearly indicate that the values of the parameters ρ and ε are significant determinants of the rate of convergence of AMLA. In fact, from Figure 4d, we can see that the selection of ρ and ε can either increase or decrease the rate of convergence of AMLA. However, it does not affect the robustness of AMLA, as AMLA is guaranteed to converge regardless of the values of ρ and ε. It is important to point out that, as seen in Figure 4d, AMLA still outperformed CGDA for the chosen values of ρ and ε. 

Overall, this proof of principle demonstrates that fully accurate and efficient computational deconvolution of tumor bulk gene expression data for estimation of the proportions of cell-type fractions, including TIICs, is best achievable using a nonlinear optimization framework, whose solution can be approximated by an accelerated machine learning algorithm (AMLA). This nonlinear optimization framework truly captures the natural state of the deconvolution problem. Consequently, in the future, we will implement the entire deconvolution workflow, described in Figure 1, as a cloud-based tool with a user-friendly graphical interface, which we shall call NECSTGEP (**N**aturally **E**stimating **C**ell-type **S**ubsets from **T**umor **G**ene **E**xpression **P**rofiles). NECSTGEP will be equipped with an additional rigorous machine learning algorithm that will be able to automatically fix the model parameters and the initialization values for AMLA, using pattern analysis of the input bulk gene expression profiles, as well as the reference profile. This is very essential as this proof of principle has shown a crucial role in yielding highly accurate estimation results. Similar problems in biology, oncology, and medical physics, such as the optimal scheduling of combined cancer therapies and reconstruction of gene regulatory networks, parade similar levels of complexity. Thus, they can benefit from an application of the technique described thus far, to yield highly accurate results. 

## 5. Conclusions

We introduced and discussed a nonlinear constrained optimization framework for the computational deconvolution of TIICs and other tumor cell-type subsets from tumor bulk gene expression profiles, in addition to an accelerated machine learning algorithm (AMLA) for directly approximating its solution. Our analysis using real tumor transcriptomics datasets concluded that this nonlinear approach yields values closely matching ground-truth values from experiment, because it treats the problem in its natural state. Models NM1 and NM2 produced the “best” values for the estimated cell-type fractions in this study and were significantly different from those obtained using the traditional linear modeling approach. However, one main limitation of the study is the empirical choice of model hyperparameters, which will be addressed in future studies. This study, therefore, heralds a paradigm shift away from the traditional linear modeling of the TIIC deconvolution problem. 

## Figures and Tables

**Table 1:** Fractions of cell types measured using flow cytometry for the lymph nodes of metastatic melanoma patients (modified from Supplementary Table S3A of [23]).

Patient ID	B Cells	CD4 T Cells	CD8 T Cells	NK Cells	Malignant Cells	Other Cells ^1^
LAU125	0.1812	0.0082	0.0035	0.0050	0.6803	0.1218
LAU355	0.3248	0.2315	0.0582	0.0017	0.0006	0.3832
LAU1255	0.0579	0.0276	0.0376	0.0017	0.3756	0.4997
LAU1314	0.4667	0.1815	0.0454	0.0025	0.0007	0.3031

^1^ These consist mostly of stromal (for example, cancer-associated fibroblasts (CAFs)) and endothelial cells.

**Table 2:** Hyperparameter values for named versions of our nonlinear framework (Equation (11)).

Version	θ	δ
ELM	1.00	1.00
LNM	0.92	1.00
NM1	0.92	1.00
NM2	1.08	1.00

**Table 3:** Fractions of cell types estimated using deconvolution methods for the lymph nodes of metastatic melanoma patients.

Patient ID	Deconvolution Method	B Cells	CD8 T Cells	CD4 T Cells	NK Cells	Macrophages	Endothelial Cells	CAF	Malignant Cells
LAU125	ELM	0.0139	0.0450	0.0714	0.0376	0.0389	0.0628	0.1175	0.6129
	LNM	0.0009	0.0170	0.0283	0.0120	0.0872	0.0468	0.1165	0.6834
	NM1	0.1884	0.0063	0.0000	0.0158	0.0684	0.0000	0.0616	0.6594
	NM2	0.1884	0.0063	0.0000	0.0158	0.0684	0.0000	0.0616	0.6594
	EPIC1 *	0.0101	0.0095	0.0303	0.0000	0.0120	0.0253	0.0003	-
	EPIC2 **	0.0000	0.0000	0.0000	0.0000	0.0258	0.0161	0.1087	0.2987
	CIBERSORTx1	0.0015	0.0000	0.0250	0.0000	0.0175	0.0002	0.0063	0.9494
	CIBERSORTx2	0.0000	0.0000	0.0107	0.0000	0.0120	0.0000	0.0135	0.9638
LAU355	ELM	0.1596	0.1154	0.3119	0.0264	0.0865	0.1008	0.0970	0.1023
	LNM	0.2558	0.1085	0.2828	0.0049	0.2287	0.0680	0.0331	0.0182
	NM1	0.3221	0.0836	0.2299	0.0084	0.0821	0.2122	0.0616	0.0000
	NM2	0.3220	0.0837	0.2300	0.0084	0.0821	0.2122	0.0616	0.0000
	EPIC1 *	0.4540	0.0182	0.2672	0.0000	0.0086	0.0000	0.0001	-
	EPIC2 **	0.1834	0.0000	0.4528	0.0000	0.1058	0.0034	0.0000	0.0000
	CIBERSORTx1	0.5550	0.0000	0.3536	0.0104	0.0794	0.0000	0.0000	0.0017
	CIBERSORTx2	0.5896	0.0000	0.3297	0.0065	0.0741	0.0000	0.0000	0.0000
LAU1255	ELM	0.0383	0.1102	0.1270	0.0390	0.0510	0.0981	0.1070	0.4292
	LNM	0.0346	0.0981	0.0985	0.0108	0.1111	0.0429	0.0610	0.5431
	NM1	0.0589	0.0521	0.0342	0.0068	0.1042	0.2974	0.1089	0.3373
	NM2	0.0589	0.0521	0.0342	0.0069	0.1042	0.2974	0.1089	0.3374
	EPIC1*	0.0411	0.1299	0.0583	0.0000	0.0197	0.0000	0.0001	-
	EPIC2**	0.0148	0.0563	0.1094	0.0000	0.0487	0.0138	0.0216	0.4628
	CIBERSORTx1	0.0493	0.0987	0.1059	0.0000	0.1360	0.0003	0.0035	0.6062
	CIBERSORTx2	0.0329	0.0993	0.0707	0.0000	0.1490	0.0002	0.0018	0.6462
LAU1314	ELM	0.1773	0.1040	0.2823	0.0343	0.0898	0.0890	0.0978	0.1257
	LNM	0.2872	0.1026	0.2519	0.0089	0.2433	0.0515	0.0284	0.0261
	NM1	0.4436	0.0452	0.1695	0.0032	0.0095	0.2506	0.0658	0.0126
	NM2	0.4436	0.0453	0.1695	0.0032	0.0094	0.2506	0.0658	0.0126
	EPIC1 *	0.6760	0.0181	0.0790	0.0042	0.0015	0.0000	0.0000	-
	EPIC2 **	0.2040	0.0001	0.4244	0.0000	0.1057	0.0045	0.0000	0.0000
	CIBERSORTx1	0.6183	0.0000	0.3229	0.0062	0.0207	0.0000	0.0000	0.0318
	CIBERSORTx2	0.6593	0.0000	0.3082	0.0099	0.0109	0.0000	0.0000	0.0115

* The reference profile does not contain malignant cells. A column labeled as “other cells” is included in the results with the values 0.9127, 0.2519, 0.7510, and 0.2212 recorded for LAU125, LAU355, LAU1255, and LAU1314, respectively. ** The results also include a column for “other cells” with values 0.5507, 0.2546, 0.2726, and 0.2613 recorded for LAU125, LAU355, LAU1255, and LAU1314, respectively.

## Data Availability

Publicly available datasets were analyzed in this study. The data can be found at https://www.ncbi.nlm.nih.gov/geo/query/acc.cgi?acc=GSE93722. The data presented in this study are openly available from https://doi.org/10.7554/eLife.26476.023, https://doi.org/10.7554/eLife.26476.024, https://doi.org/10.1038/s41587-019-0114-2.

## Appendix A

Let C be a nonempty, closed, and convex subset of a Hilbert space ℍ.

**Definition** **1.** *A vector*$v:={℘}_{C}\left(u\right)\in C$*is called the projection of*u∈ℍ onto C *if and only if* $v=\underset{z\in C}{\inf}\|u-z{\|}^{2}$. *Equivalently, if and only if* 〈v−u, v−z〉≤0 ∀ z∈C.

**Definition** **2.** *A mapping*Γ*(a) is said to be monotone on*C*if and only if*$\langle \Gamma u-\Gamma v,\;u-v\rangle \geq 0\;\forall \;\left(u,v\right)\in C\times C$. *Furthermore, if there exists*ξ>0*such that*$\langle \Gamma u-\Gamma v,\;u-v\rangle \geq \xi \|u-v{\|}^{2},\;\forall \;\left(u,v\right)\in C\times C$, *then*Γ*is called*ξ*-strongly monotone. (b) is said to be Lipschitz on*C, *if there exists a scalar*K>0*such that*‖Γu−Γv‖≤K‖u−v‖, $\forall \;\left(u,v\right)\in C\times C.\;$*A monotone operator is said to be maximal monotone if it has no proper monotone extension.*

**Definition** **3.** *A subset*S *of*ℍ*is said to be bounded if and only if there exists a scalar*μ>0*such that*‖u‖≤μ ∀ u∈S.

**Definition** **4.** *Let*g*be a differentiable mapping on*C. *A vector*$u^{*}\in \;C$*is called a stationary (critical) point of*g*if*$\langle \nabla_{g}\left(u^{*}\right),u-u^{*}\rangle \geq 0$∀ u∈C.

**Definition** **5.** *Let*$g:{\mathbb{R}}^{n}\to \mathbb{R}\cup \left\{+\infty \right\}$*be a proper lower semicontinuous mapping and*∂g*denote the subdifferential of*g . g *is said to have the Kurdyka–Lojasiewicz (KL) property at* $u^{*}\in dom\partial g=\left\{u\in {\mathbb{R}}^{n}:\partial g\left(u\right)\neq \phi \right\}$, *if there exists* $\eta \in \left(0,+\infty \right]$, *a neighborhood* V *of* $u^{*}$, *and a continuous concave map* $\varphi :\left[0,\eta \right)\to {\mathbb{R}}^{+}$ *with* $\varphi \left(0\right)=0$, φ *is* ${∁}^{1}$ *on* $\left(0,\eta \right)$, $\varphi \prime \left(s\right)>0\;\forall \;s\in \left(0,\eta \right)$ *and* $\varphi \prime \left(g\left(u\right)-g\left(u^{*}\right)\right)\;dist\left(0,\partial g\left(u\right)\right)\geq 1\;\forall \;u\in V\cap \left\{v\in {\mathbb{R}}^{n}:\;g\left(u^{*}\right)<g\left(v\right)<g\left(u^{*}\right)+\eta \right\}$, *where* $dist\left(z,S\right)=\underset{s\prime \in \;S}{\inf}\|z-s\prime {\|}^{2}$. *Any* g *satisfying the KL property at each point of* dom∂g, *is called the KL function. The set of KL functions is rich, including as subsets, real and sub-analytic functions, real semi-algebraic functions, semi-convex functions, uniformly convex functions, and convex functions satisfying a growth condition (see* [51,52,53] *and references contained therein).*

**Lemma** **6.** *Let*g*be a differentiable mapping on*C*with*K*-Lipschitz gradient*$\nabla_{g}$; *then*, $\left|g\left(u\right)-g\left(v\right)-\langle u-v,\nabla_{g}\left(v\right)\rangle \right|\leq \frac{K}{2}\|u-v{\|}^{2}\;\forall \;u,v\in C$.

**Proof.** Let $g_{0}$ be a real valued map on ℝ defined by $g_{0}\left(t\right)=g\left(v+t\left(u-v\right)\right)$; then, it follows that $g_{0}\left(0\right)=g\left(v\right),\;g_{0}\left(1\right)=g\left(u\right)$ and

$$g\left(u\right)-g\left(v\right)=g_{0}\left(1\right)-g_{0}\left(0\right)=\int_{0}^{1}\;g_{0}\prime \left(t\right)dt.$$

Now, $g_{0}\prime \left(t\right)=\langle u-v,\;\nabla_{g}\left(v+t\left(u-v\right)\right)\rangle$; thus,

$$\begin{array}{cc} |g(u)-g(v)\;- & \langle u-v,\nabla_{g}\left(v\right)\rangle |\\ \; & =\left|\int_{0}^{1}\langle u-v,\nabla_{g}\left(v+t\left(u-v\right)\right)\rangle dt-\langle u-v,\nabla_{g}\left(v\right)\rangle \right|\\ \; & \leq \int_{0}^{1}\left|\langle u-v,\nabla_{g}\left(v+t\left(u-v\right)\right)-\nabla_{g}\left(v\right)\rangle \right|dt\\ \; & \leq \int_{0}^{1}∥\nabla_{g}\left(v+t\left(u-v\right)\right)-\nabla_{g}\left(v\right)∥∥u-v∥dt\\ \; & \leq \int_{0}^{1}{Kt∥u-v∥}^{2}dt=\frac{K}{2}{∥u-v∥}^{2}.□ \end{array}$$

**Lemma** **7.** [54] *Let* ${\left\{u_{n}\right\}}_{n\geq 0}\subseteq ℍ$ *be a bounded sequence in* ℍ*. Then, there exists a subsequence* ${\left\{u_{n_{j}}\right\}}_{j\geq 0}\subseteq {\left\{u_{n}\right\}}_{n\geq 0}$ *that converges weakly (denoted as* $u_{n_{j}}⇀u_{*}$ *(say)) in* ℍ*. Note that, for* $ℍ={\mathbb{R}}^{N}$*, strong and weak convergence coincide.*

**Lemma** **8.** *Let*${\left\{u_{n}\right\}}_{n\geq 0}$*and*${\left\{z_{n}\right\}}_{n\geq 0}$*be real sequences such that*$u_{n}\geq 0\;\forall \;n\geq 0$, $\underset{n\to \infty }{\lim}\overset{n}{\underset{i=0}{\sum }}z_{i}\in \mathbb{R}$*and*$u_{n+1}\leq u_{n}+z_{n}\;\forall \;n\geq 0$*. Then*$\underset{n\to \infty }{\lim}u_{n}$*exists in*ℝ.

 **Proof.** We follow a method of proof in [55]. Let, $\underset{n\to \infty }{\lim}\overset{n}{\underset{i=0}{\sum }}z_{i}=z_{*}$. Define $y_{n}=\overset{n-1}{\underset{i=0}{\sum }}z_{i}\;\forall \;n\geq 1$; then, $\underset{n\to \infty }{\lim}y_{n}=z_{0}$. Now, $u_{n+1}+y_{n}\leq u_{n}+z_{n}+y_{n}=u_{n}+y_{n+1}\;\forall \;n\geq 1$. Thus, $u_{n+1}-y_{n+1}\leq$ $u_{n}-y_{n}\;\forall \;n\geq 1$. Therefore, the sequence ${\left\{u_{n}-y_{n}\right\}}_{n\geq 1}$ is monotone nonincreasing. Hence, only the following two cases are possible: Case 1: $\underset{n\to \infty }{\lim}\left(u_{n}-y_{n}\right)=-\infty ,$ Case 2: $\underset{n\to \infty }{\lim}\left(u_{n}-y_{n}\right)\in \;\mathbb{R}$. It is impossible for case 1 to be true because of the following contradiction: Assuming case 1 holds and using the hypothesis that $u_{n}\geq 0\;\forall \;n\geq 1\;$,

$$0\leq \underset{n\to \infty }{\lim}u_{n}=\underset{n\to \infty }{\lim}\left(\left(u_{n}-y_{n}\right)+y_{n}\right)=-\infty .$$

Hence, case 2 must be true. Consequently, we have that

$$\underset{n\to \infty }{\lim}u_{n}=\underset{n\to \infty }{\lim}\left(\left(u_{n}-y_{n}\right)+y_{n}\right)=\underset{n\to \infty }{\lim}\left(u_{n}-y_{n}\right)+\underset{n\to \infty }{\lim}y_{n}\;\in \mathbb{R}.\;□\;$$

**Lemma** **9.** [56] *Let* Γ *be a single valued monotone operator on* ℍ *such that* $C\subseteq \;dom\Gamma =\left\{u\in ℍ:\Gamma \left(u\right)\neq \phi \right\}$ *and* Γ *is hemicontinuous on* C*. Let* $T_{C}$ *be the normality operator for* C*, i.e.,* $T_{C}\left(u\right)=\left\{y\in ℍ:\;\langle u-v,\;y\rangle \geq 0,\;\forall \;v\in C\right\}.\;$*Then,* $\Gamma +T_{C}$ *is a maximal monotone operator.*

**Lemma** **10.** [57] *Let* ${\left\{u_{n}\right\}}_{n\geq 0}$ *be a sequence in* ℍ *that converges weakly to* $u_{0}\in ℍ.\;$*Then, for any* $u\neq u_{0},\;\underset{n\to 0}{\lim}\inf\|u_{n}-u_{0}\|<\underset{n\to 0}{\lim}\inf\|u_{n}-u\|.$

**Lemma** **11.** [58] *Let* $g:{\mathbb{R}}^{2N}\to \mathbb{R}\cup \left\{+\infty \right\}$ *(sic) be a proper lower semicontinuous mapping and* $z_{n}={\left(u_{n},\;u_{n-1}\right)}_{n\geq 1}$ *be a sequence satisfying the following:*

 *(H1)* *for each* n≥1, $g\left(z_{n+1}\right)+a\|u_{n}-u_{n-1}\|{}^{2}\leq g\left(z_{n}\right)$*for some fixed positive constant*a;

 *(H2)* *for each*n≥1*, there exists*$y_{n+1}\in \partial g\left(z_{n+1}\right)$*such that*$\|y_{n+1}\|\leq \frac{b}{2}\left(\left\|u_{n}-u_{n-1}\|+\|u_{n+1}-u_{n}\right\|\right)$*for some fixed positive constant*b;

 *(H3)* *there exists a subsequence*${\left(z_{n_{k}}\right)}_{k\geq 1}$*such that*$z_{n_{k}}\to \hat{z}$*and*$g\left(z_{n_{k}}\right)\to g\left(\hat{z}\right)$*as*k→∞.

*Moreover, let*g*have the KL property at the cluster point*$\hat{z}$*specified in (H3). Then the sequence*${\left\{u_{n}\right\}}_{n\geq 0}$*has finite length (that is*$\overset{\infty }{\underset{n=1}{\sum }}\|u_{n}-u_{n-1}\|<+\infty$) *and converges to*$\hat{u}$*as*n→∞, *where*$\left(\hat{u},\;\hat{u}\right)$*is a critical point of*g.

**Theorem** **12.**
*Let* g *be a real valued mapping on* C *, bounded below, and whose Frechet derivative denoted by* A *is* K *-Lipschitz. Let* ${\left\{v_{j}\right\}}_{j\geq 0}$ *be the sequence generated iteratively by AMLA (see Equation (12)), where the control parameters* $\alpha_{j}$ *and* λ *are chosen in* ℝ *such that*

$0\leq \alpha_{j}<\frac{2-\lambda K}{3},\;\underset{j\to \infty }{\lim}\alpha_{j}=0,\;\alpha_{j+1}\leq \alpha_{j}\;\forall \;j\geq 0\;and\;0<\lambda <\frac{1}{K}.\;Then,\;$

(a) *there exists* μ,α>0*such that for the sequence *${\left\{f\left(v_{j}\right)\right\}}_{j\geq 0}={\left\{g\left(v_{j}\right)+\mu \|v_{j}-v_{j-1}\|{}^{2}\right\}}_{j\geq 0}$*,*$f\left(v_{j+1}\right)+\alpha \|v_{j+1}-v_{j}\|{}^{2}\leq \;f\left(v_{j}\right)\;\forall \;j\geq 0;$
(b) $\overset{\infty }{\underset{j=0}{\sum }}\|v_{j+1}-v_{j}\|{}^{2}<+\infty ;$
(c) $\underset{j\to \infty }{\lim}g\left(v_{j}\right)\in \mathbb{R}.$

 **Proof.** According to Lemma 6, we have that $g\left(v_{j+1}\right)-g\left(v_{j}\right)\leq v_{j+1}-v_{j},\;Av_{j}+\frac{K}{2}v_{j+1}-v_{j}{}^{2}$. This implies that

$$\begin{array}{c} \lambda \left(g\left(v_{j+1}\right)-g\left(v_{j}\right)\right)\leq \langle v_{j+1}-v_{j},\lambda Av_{j}\rangle +\frac{\lambda K}{2}{∥v_{j+1}-v_{j}∥}^{2}\\ =\langle v_{j+1}-v_{j},w_{j}-\left(w_{j}-\lambda Av_{j}\right)\rangle +\frac{\lambda K}{2}{∥v_{j+1}-v_{j}∥}^{2}\\ =\langle v_{j+1}-v_{j},v_{j+1}-\left(w_{j}-\lambda Av_{j}\right)\rangle +\langle v_{j+1}-v_{j},w_{j}-v_{j+1}\rangle +\frac{\lambda K}{2}{∥v_{j+1}-v_{j}∥}^{2}\\ \leq -\left(1-\frac{\lambda K}{2}\right){∥v_{j+1}-v_{j}∥}^{2}+\alpha_{j}\langle v_{j+1}-v_{j},v_{j-1}-v_{j}\rangle \\ \leq -\left(1-\frac{\lambda K}{2}\right){∥v_{j+1}-v_{j}∥}^{2}+\alpha_{j}\langle v_{j+1}-v_{j},v_{j-1}-v_{j}\rangle +\frac{\alpha_{j}}{2}{∥v_{j+1}-v_{j-1}∥}^{2}\\ =-\left(1-\frac{\lambda K}{2}\right){∥v_{j+1}-v_{j}∥}^{2}+\frac{\alpha_{j}}{2}{∥v_{j+1}-v_{j}∥}^{2}+\frac{\alpha_{j}}{2}{∥v_{j}-v_{j-1}∥}^{2}\\ =-\left(1-\frac{\lambda K+\alpha_{j}}{2}\right){∥v_{j+1}-v_{j}∥}^{2}+\frac{\alpha_{j}}{2}{∥v_{j}-v_{j-1}∥}^{2}\\ \;\leq -\left(\frac{2-\lambda K}{3}\right){∥v_{j+1}-v_{j}∥}^{2}+\left(\frac{2-\lambda K}{6}\right){∥v_{j}-v_{j-1}∥}^{2} \end{array}$$

Thus, we have that

$$\begin{array}{c} \lambda \left(g\left(v_{j+1}\right)-g\left(v_{j}\right)\right)+\left(\frac{2-\lambda K}{6}\right)\left({∥v_{j+1}-v_{j}∥}^{2}-{∥v_{j}-v_{j-1}∥}^{2}\right)\\ \leq -\left(\frac{2-\lambda K}{6}\right){∥v_{j+1}-v_{j}∥}^{2}. \end{array}$$

Define the sequence ${\left\{f\left(v_{j+1}\right)\right\}}_{j\geq 1}$ as

$$\;f\left(v_{j}\right)=g\left(v_{j}\right)+\left(\frac{2-\lambda K}{6\lambda }\right)\|v_{j}-v_{j-1}\|{}^{2}.$$

Then, ${\left\{f\left(v_{j+1}\right)\right\}}_{j\geq 1}$ is bounded below, and it follows that

$$f\left(v_{j+1}\right)+\alpha \|v_{j+1}-v_{j}\|{}^{2}\leq \;f\left(v_{j}\right)\;\forall \;j\geq 1\;where\;\alpha =\frac{2-\lambda K}{6\lambda }.\;$$

Thus, the sequence is monotone nonincreasing and bounded below. Hence, $\underset{j\to \infty }{\lim}f\left(v_{j}\right)$ exists. Moreover, we have that

$$\overset{\infty }{\underset{j=0}{\sum }}\left(\frac{2-\lambda K}{6\lambda }\right)\|v_{j+1}-v_{j}\|{}^{2}<+\infty .\;$$

Therefore,

$$\overset{\infty }{\underset{j=0}{\sum }}\|v_{j+1}-v_{j}\|{}^{2}<+\infty ,\;\underset{j\to \infty }{\lim}\|v_{j+1}-v_{j}\|{}^{2}=0\;and\;\underset{j\to \infty }{\lim}g\left(v_{j}\right)\;exists.\;□\;$$

**Theorem** **13.** 
*Let the assumptions of Theorem 12 hold. Suppose further that* A *is monotone; denote by* $S_{Cg}$ *the set of critical points of* g *. Let* $v^{*}\in S_{Cg}$ *and* ${\left\{v_{j}\right\}}_{j\geq 0}$ *be the sequence generated by AMLA. Then, the following applies:*

- ${\left\{v_{j}\right\}}_{j\geq 0}$ *is bounded;*
- $\underset{j\to \infty }{\lim}\|v_{j+1}-w_{j}\|{}^{2}=0;$
- $\underset{j\to \infty }{\lim}\|v_{j}-v^{*}\|{}^{2}$ *exists;*
- *the sequence*${\left\{v_{j}\right\}}_{j\geq 0}$*converges waekly to a critical point*$\;\hat{v}$*of* g*; moreover, if* g*is convex, then* $\hat{v}$*is a minimizer of* g

 **Proof.** Let $u_{j}\;:=w_{j}-\lambda Av_{j}$ and $\tau_{j}\;:=2\lambda \left(g\left(v_{j}\right)-g\left(v_{j+1}\right)\right)+\lambda K\|v_{j+1}-v_{j}\|{}^{2}$. Now, using the definition of $v_{j}$ and $w_{j}$, Definition 1, the fact that $v^{*}\in S_{Cg}$, and the monotonicity of A, we get the following estimation:

(A1)
$$\begin{array}{c} {∥v_{j+1}-v^{*}∥}^{2}\leq {∥u_{j}-v^{*}∥}^{2}-{∥v_{j+1}-u_{j}∥}^{2}\\ ={∥w_{j}-\lambda Av_{j}-v^{*}∥}^{2}-{∥v_{j+1}-\left(w_{j}-\lambda Av_{j}\right)∥}^{2}\\ ={∥w_{j}-v^{*}∥}^{2}-2\lambda \langle w_{j}-v^{*},Av_{j}\rangle +{∥\lambda Av_{j}∥}^{2}\\ -\left({∥v_{j+1}-w_{j}∥}^{2}+2\lambda \langle v_{j+1}-w_{j},Av_{j}\rangle +{∥\lambda Av_{j}∥}^{2}\right)\\ ={∥w_{j}-v^{*}∥}^{2}-2\lambda \langle v_{j+1}-v^{*},Av_{j}\rangle -{∥v_{j+1}-w_{j}∥}^{2}\\ \leq {∥w_{j}-v^{*}∥}^{2}-2\lambda \langle v_{j+1}-v_{j},Av_{j}\rangle -{∥v_{j+1}-w_{j}∥}^{2}\\ \leq \left(1-\alpha_{j}\right){∥v_{j}-v^{*}∥}^{2}+\alpha_{j}{∥v_{j-1}-v^{*}∥}^{2}-2\lambda \langle v_{j+1}-v_{j},Av_{j}\rangle . \end{array}$$

Next, according to Lemma 6,

$$-2\lambda \langle v_{j+1}-v_{j},Av_{j}\rangle \leq 2\lambda \left(g\left(v_{j}\right)-g\left(v_{j+1}\right)\right)+\lambda K\|v_{j+1}-v_{j}\|{}^{2}.\;$$

Thus,

$$\begin{array}{c} {∥v_{j+1}-v^{*}∥}^{2}\leq {∥v_{j}-v^{*}∥}^{2}-\alpha_{j}\left({∥v_{j}-v^{*}∥}^{2}-{∥v_{j-1}-v^{*}∥}^{2}\right)\\ +2\lambda \left(g\left(v_{j}\right)-g\left(v_{j+1}\right)\right)+\lambda K{∥v_{j+1}-v_{j}∥}^{2}\\ \leq {∥v_{j-1}-v^{*}∥}^{2}-\alpha_{j-1}\left({∥v_{j-1}-v^{*}∥}^{2}-{∥v_{j-2}-v^{*}∥}^{2}\right)+\tau_{j-1}\\ -\alpha_{j}\left({∥v_{j}-v^{*}∥}^{2}-{∥v_{j-1}-v^{*}∥}^{2}\right)+\tau_{j}\\ \vdots \\ \leq {∥v_{1}-v^{*}∥}^{2}+\sum_{i=1}^{j}-\alpha_{i}\left({∥v_{i}-v^{*}∥}^{2}-{∥v_{i-1}-v^{*}∥}^{2}\right)+\sum_{i=1}^{j}\tau_{i}\\ \leq {∥v_{1}-v^{*}∥}^{2}+\alpha_{1}{∥v_{0}-v^{*}∥}^{2}-\alpha_{j}{∥v_{j}-v^{*}∥}^{2}+\sum_{i=1}^{j}\tau_{i}\\ \leq {∥v_{1}-v^{*}∥}^{2}+\alpha_{1}{∥v_{0}-v^{*}∥}^{2}+M,\forall j\geq 1.\; \end{array}$$

Therefore, ${\left\{v_{j}\right\}}_{j\geq 0}$ is bounded.

Next, $\|v_{j+1}-v^{*}\|{}^{2}\leq \|v_{j}-v^{*}\|{}^{2}-\alpha_{j}\left(\|v_{j}-v^{*}\|{}^{2}-\|v_{j-1}-v^{*}\|{}^{2}\right)+\tau_{j}$, such that,

(A2)
$$\|v_{j+1}-v^{*}\|{}^{2}+\alpha_{j}\|v_{j}-v^{*}\|{}^{2}\leq \|v_{j}-v^{*}\|{}^{2}+\alpha_{j-1}\|v_{j-1}-v^{*}\|{}^{2}+\tau_{j}.\;$$

According to Lemma 8, we have that $\underset{j\to \infty }{\lim}\left(\|v_{j}-v^{*}\|{}^{2}+\alpha_{j-1}\|v_{j-1}-v^{*}\|{}^{2}\right)\;$ exists.

Moreover, combining Equations (A1) and (A2) and Theorem 12(b) yields $\underset{j\to \infty }{\lim}\|v_{j+1}-w_{j}\|{}^{2}=0\;$ and $\underset{j\to \infty }{\lim}\|v_{j}-w_{j}\|=0$.

Since $\underset{j\to \infty }{\lim}\alpha_{j}=0$ and ${\left\{\|v_{j}-v^{*}\|{}^{2}\right\}}_{j\geq 0}$ is bounded, then $\underset{j\to \infty }{\lim}\alpha_{j-1}\|v_{j-1}-v^{*}\|{}^{2}=0$.

Hence, $\underset{j\to \infty }{\lim}\|v_{j}-v^{*}\|{}^{2}$ exists.

Now, we show that every weak sequential limit of ${\left\{v_{j}\right\}}_{j\geq 0}$ is in $S_{Cg}$. Let $W_{s}\left(v_{j}\right)$ denote the set of weak sequential limits of ${\left\{v_{j}\right\}}_{j\geq 0}$. Then, the boundedness of ${\left\{v_{j}\right\}}_{j\geq 0}$ guarantees that $W_{s}\left(v_{j}\right)$ is nonempty (see Lemma 7). Let $\hat{v}\in W_{s}\left(v_{j}\right)$; given Definition 4, it suffices to show that $\langle v-\hat{v},\;A\hat{v}\rangle \geq 0\;\forall \;v\in C$. By definition of $W_{s}\left(v_{j}\right)$, there exists a subsequence ${\left\{v_{j_{k}}\right\}}_{k\geq 0}\subseteq {\left\{v_{j}\right\}}_{j\geq 0}$ such that $v_{j_{k}}⇀\hat{v}$. $\|v_{j}-w_{j}\|⟶0⟹w_{j_{k}}⇀\hat{v}$.

Define $G\left(v\right)=\left\{\begin{array}{c} Av+T_{C}\left(v\right),\;if\;v\in C\\ \phi ,\;if\;v\notin C \end{array}\right.$, where $T_{C}$ is the normality operator. According to Lemma 9, G is maximal monotone and $0\in \;G\left(v\right)$ if and only if $\left(v\in S_{Cg}\right)$. Thus, it suffices to show that $\left(\hat{v},\;0\right)\in \;graph\left(G\right)=\left\{\left(v,u\right)\in ℍ\times ℍ:v\in \;dom\;\left(G\right),\;u\in G\left(v\right)\right\}$.

We recall that, for any maximal monotone operator Γ, if 〈Γx−z, x−y〉≥0 ∀ x∈ dom Γ and z∈ℍ, then $z\in \Gamma \left(y\right)$.

Thus, let $\left(v,u^{*}\right)\in \;graph\left(G\right)$ be arbitrary, $\langle u^{*},\;v-\hat{v}\rangle \geq 0⟹0\in G\left(\hat{v}\right)$. Hence, we only need to verify that $\langle u^{*},\;v-\hat{v}\rangle \geq 0$.

Now, $\left(v,u^{*}\right)\in \;graph\left(G\right)\;⟹\;u^{*}-Av\;$ $\in T_{C}\left(v\right)⟹\langle u^{*}-Av,\;v-u\rangle \geq 0\;\forall \;u\in C$.

However, by definition, $v_{j+1}={℘}_{C}\left(w_{j}-\lambda Av_{j}\right)$, thus, $\langle v_{j+1}-v,\;w_{j}-\lambda Av_{j}-v_{j+1}\rangle \geq 0\;⟹\langle v-v_{j+1},\frac{v_{j+1}-w_{j}}{\lambda }+Av_{j}\rangle \geq 0.$

Since $v_{j}\in C\;\forall \;j\geq 0$, we have that

$$\begin{array}{c} \langle v-v_{j_{k}},u^{*}\rangle \geq \langle v-v_{j_{k}},Av\rangle \geq \langle v-v_{j_{k}},Av\rangle -\langle v-v_{j_{k}}\frac{v_{j_{k}}-w_{j_{k}-1}}{\lambda }+Av_{j_{k}-1}\rangle \\ =\langle v-v_{j_{k}},Av-Av_{j_{k}}\rangle +\langle v-v_{j_{k}},Av_{j_{k}}-Av_{j_{k}-1}\rangle +\frac{1}{\lambda }\langle v-v_{j_{k}},w_{j_{k}-1}-v_{j_{k}}\rangle . \end{array}$$

Taking limit as k→∞, we get $\langle u^{*},\;v-\hat{v}\rangle \geq 0$. Therefore, $\hat{v}\in S_{Cg}$ and $W_{s}\left(v_{j}\right)\subseteq S_{Cg}.$

Finally, we show that $v_{j}⇀v\prime \in S_{Cg}$. To verify this, it is enough to show that $W_{s}\left(v_{j}\right)$ is a singleton. We proceed by contradiction. Suppose there exist $v^{\prime },\;v\prime \prime \in W_{s}\left(v_{j}\right)$ with $v^{\prime }\neq \;v\prime \prime$, then there are subsequences ${\left\{v_{j_{k}}\right\}}_{k\geq 0},{\left\{v_{j_{i}}\right\}}_{i\geq 0}\;$ of ${\left\{v_{j}\right\}}_{j\geq 0}$ such that $v_{j_{k}}⇀v\prime$ and $v_{j_{i}}⇀v^{\prime \prime }$. Therefore, according to Lemma 10, we have that

$$\begin{array}{c} \lim_{j\to \infty }∥v_{j}-v^{\prime }∥=\underset{k\to \infty }{\mathrm{liminf}}∥v_{j_{k}}-v^{\prime }∥<\underset{k\to \infty }{\mathrm{liminf}}∥v_{j_{k}}-v^{\prime \prime }∥=\lim_{j\to \infty }∥v_{j}-v^{\prime \prime }∥\\ =\underset{i\to \infty }{\mathrm{liminf}}∥v_{j_{i}}-v^{\prime \prime }∥<\underset{i\to \infty }{\mathrm{limin}f}∥v_{j_{i}}-v^{\prime }∥=\lim_{j\to \infty }∥v_{j}-v^{\prime }∥. \end{array}$$

This is a contradiction since $\underset{j\to \infty }{\lim}\|v_{j}-v^{\prime }\|$ is never less than itself.

Hence, the sequence ${\left\{v_{j}\right\}}_{j\geq 0}$ converges weakly to a critical point of g. Furthermore, if g is convex, then, from $0\leq g\left(v\right)-g\left(\hat{v}\right)-\langle v-\hat{v},A\hat{v}\rangle \;$ and $\langle v-\hat{v},A\hat{v}\rangle \geq 0\;\forall \;v\in C$, we obtain $g\left(\hat{v}\right)\leq g\left(v\right)\;\forall \;v\in C$. Thus, $\hat{v}$ is a minimizer of g. □

**Theorem** **14.** *Let the assumptions of Theorem 12 hold for*$ℍ={\mathbb{R}}^{n}$*. In addition, assume further that*C*is bounded and*g*possesses the KL property. Then, the sequenc*e ${\left\{v_{j}\right\}}_{j\geq 0}$*generated by AMLA converges to an element of*$S_{Cg}$.

 **Proof.** By definition, $v_{j}\in C\;\forall \;j\geq 0$. Hence, the sequence ${\left\{v_{j}\right\}}_{j\geq 0}$ is bounded, and $W_{s}\left(v_{j}\right)\neq \phi .$ We now show that $W_{s}\left(v_{j}\right)\subseteq S_{Cg}$. Let $\hat{v}\in W_{s}\left(v_{j}\right)$ be arbitrary; then, there is a subsequence ${\left\{v_{j_{k}}\right\}}_{k\geq 0}$ of ${\left\{v_{j}\right\}}_{j\geq 0}$ such that $v_{j_{k}}\to \hat{v}$ as k→∞. Using Theorem 12(b) and the definition of $w_{j}$, we get $\underset{j\to \infty }{\lim}\|v_{j+1}-v_{j}\|=\underset{j\to \infty }{\lim}\|v_{j+1}-w_{j}\|=\underset{j\to \infty }{\lim}\|v_{j}-w_{j}\|=0$. Therefore, $\underset{k\to \infty }{\lim}v_{j_{k}}-w_{j_{k}}=\underset{k\to \infty }{\lim}\|v_{j_{k}+1}-w_{j_{k}}\|=\underset{k\to \infty }{\lim}\|v_{j_{k}+1}-v_{j_{k}}\|=0$. This implies that $w_{j_{k}}\to \hat{v}$ and $v_{j_{k}+1}\to \hat{v}$ as k→∞. Furthermore, the continuity of A guarantees that $Av_{j_{k}}\to A\hat{v}$ as k→∞. Now, let v∈C be arbitrary; then, by definition of $v_{j_{k}+1}$, $\langle v_{j_{k}+1}-v,\;w_{j_{k}}-\lambda Av_{j_{k}}-v_{j_{k}+1}\rangle \geq 0$

$$⟹\;\langle v-v_{j_{k}+1},\;\lambda Av_{j_{k}}\rangle +\langle v-v_{j_{k}+1},\;v_{j_{k}+1}-w_{j_{k}}\rangle \geq 0.$$

Taking the limit as k→∞, we have that$\langle \;v-\hat{v},\;\lambda A\hat{v}\rangle \geq 0\;⟹\langle v-\hat{v},\;A\hat{v}\rangle \geq 0\;\forall \;\in C$. Hence, $W_{s}\left(v_{j}\right)\subseteq S_{Cg}$.

Next, we make use of Lemma 11 to prove that $v_{j}\to \hat{v}$. For this, we define the map $h:{\mathbb{R}}^{n}\times {\mathbb{R}}^{n}\to \mathbb{R}\cup \left\{+\infty \right\}$as follows:

$$h\left(x,y\right)=g\left(x\right)+\omega \|x-y{\|}^{2}+I_{C}\left(x\right)≔F\left(x,y\right)+I_{C}\left(x\right),$$

where $I_{C}\left(x\right)=\left\{\begin{array}{c} 0\;,\;if\;x\in C\\ +\infty \;,\;otherwise \end{array}\right.$; then, h has the KL property on $dom\left(h\right)$.

Set ${\left(x_{j}\right)}_{j\geq 1}={\left(v_{j},v_{j-1}\right)}_{j\geq 1}$; then, Theorem 12(a) gives that H1 of Lemma 11 holds. Moreover, the boundedness of ${\left\{v_{j}\right\}}_{j\geq 0}$ and continuity of h on C imply that there exists a subsequence ${\left(x_{jk}\right)}_{k\geq 1}$ of ${\left(x_{j}\right)}_{j\geq 1}$ such that $x_{jk}\to \hat{x}=\left(\hat{v},\hat{v}\right)$ and $h\left(x_{jk}\right)\to h\left(\hat{x}\right)$, as k→∞, thus verifying H3 of Lemma 11.

Next, we show that H2 of Lemma 11 also holds. First, we recall that

$$\begin{array}{c} \left(u_{m}^{1},u_{m}^{2}\right)=u_{m}\in \partial h\left(x_{m}\right)=\left(\nabla g\left(v_{m}\right)+2\omega \left(v_{m}-v_{m-1}\right)+T_{C}v_{m},2\omega \left(v_{m-1}-v_{m}\right)\right)\\ ⟺u_{m}^{1}\in \nabla g\left(v_{m}\right)+2\omega \left(v_{m}-v_{m-1}\right)+T_{C}v_{m}\cdot \;\mathrm{and}\;\cdot u_{m}^{2}=2\omega \left(v_{m-1}-v_{m}\right)\\ ⟺u_{m}^{1}-\nabla g\left(v_{m}\right)-2\omega \left(v_{m}-v_{m-1}\right)\in T_{C}v_{m}\cdot \;\mathrm{and}\;\cdot u_{m}^{2}=2\omega \left(v_{m-1}-v_{m}\right)\\ ⟺u_{m}^{1}-Av_{m}-2\omega \left(v_{m}-v_{m-1}\right)\in T_{C}v_{m}\cdot \;\mathrm{and}\;\cdot u_{m}^{2}=2\omega \left(v_{m-1}-v_{m}\right)\\ ⟺\langle v_{m}-y,u_{m}^{1}-Av_{m}-2\omega \left(v_{m}-v_{m-1}\right)\rangle \geq 0\forall y\in C\;\mathrm{and}\;\cdot u_{m}^{2}=2\omega \left(v_{m-1}-v_{m}\right). \end{array}$$

Now, by definition of $v_{m+1}$, we have $\langle v_{m+1}-y,\;w_{m}-\lambda Av_{m}-v_{m+1}\rangle \geq 0\;\forall \;y\in C$. Thus,

$$\begin{array}{c} \langle v_{m+1}-y,\frac{w_{m}-v_{m+1}}{\lambda }-Av_{m}\rangle \geq 0\forall y\in C\\ \Leftrightarrow \langle v_{m+1}-y,\frac{w_{m}-v_{m+1}}{\lambda }+Av_{m+1}-Av_{m}-Av_{m+1}\rangle \geq 0\forall y\in C\\ \Leftrightarrow \langle v_{m+1}-y,\frac{w_{m}-v_{m+1}}{\lambda }+Av_{m+1}-Av_{m}+2\omega \left(v_{m+1}-v_{m}\right)-Av_{m+1}-2\omega \left(v_{m+1}-v_{m}\right)\rangle \geq 0\forall y\in C\\ \Rightarrow u_{m}=\left(\frac{w_{m}-v_{m+1}}{\lambda }+Av_{m+1}-Av_{m}+2\omega \left(v_{m+1}-v_{m}\right),2\omega \left(v_{m}-v_{m+1}\right)\right)\in \partial h\left(x_{m+1}\right). \end{array}$$

An estimation using the definition of $w_{m}$ and Lipschitz continuity of A gives

$$\begin{array}{c} ∥u_{m}∥\leq ∥\frac{w_{m}-v_{m+1}}{\lambda }+Av_{m+1}-Av_{m}+2\omega \left(v_{m+1}-v_{m}\right)∥+∥2\omega \left(v_{m}-v_{m+1}\right)∥\\ \leq \left(4\omega +K+\frac{1}{\lambda }\right)\left(∥v_{m+1}-v_{m}∥+∥v_{m}-v_{m-1}∥\right). \end{array}$$

Therefore, Lemma 11 guarantees that $x_{j}⟶\hat{x}=\left(\hat{v},\hat{v}\right)$. Since $x_{j}=\left(v_{j},v_{j-1}\right)$, we have that $v_{j}⟶\hat{v}\in W_{s}\left(v_{j}\right)\subseteq S_{Cg.}\;□$
